# Supplementary figures and images for: The inside scoop: Comparative genomics of two intranuclear bacteria, “Candidatus Berkiella cookevillensis” and “Candidatus Berkiella aquae”
Source: PLoS One. 2022 Dec 30;17(12):e0278206. doi: 10.1371/journal.pone.0278206 (PMC9803151; doi:10.1371/journal.pone.0278206)

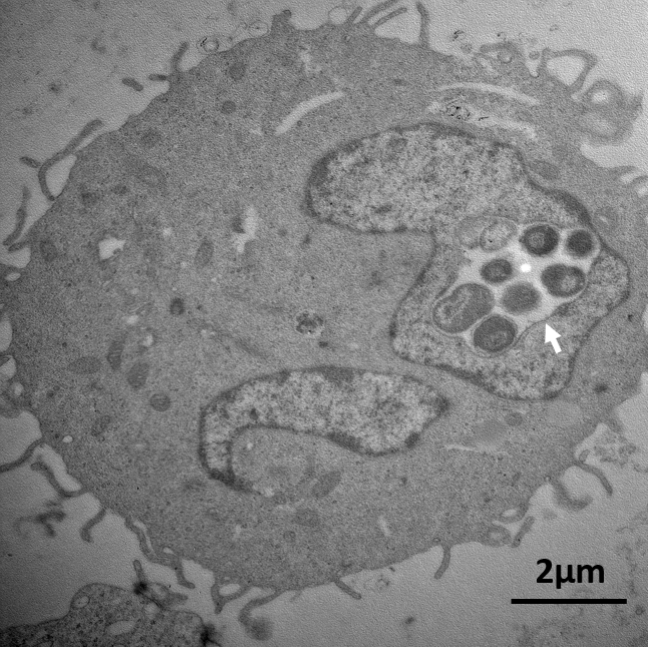

Supplement: S1 Fig — Transmission electron micrograph of Thp1 cell infected with CC99. CC99-CV appears within the nucleus at 24-hour post infection (12,000x magnification). (TIF) [file pone.0278206.s001.tif]
