# Supplementary material for: The inside scoop: Comparative genomics of two intranuclear bacteria, “Candidatus Berkiella cookevillensis” and “Candidatus Berkiella aquae”
Source: PLoS One. 2022 Dec 30;17(12):e0278206. doi: 10.1371/journal.pone.0278206 (PMC9803151; doi:10.1371/journal.pone.0278206)
Supplement: S2 Table — (DOCX) [file pone.0278206.s003.docx]

**Table S2.** Genes Involved in Amino Acid Biosynthesis Reactions

|  | **Genes** | **“*Ca.* B. cookevillensis” (CC99)** | **“*Ca.* B. aquae” (HT99)** |
| --- | --- | --- | --- |
| **L-Alanine** | *iscS* - Cysteine desulfurase (EC 2.8.1.7) | fig\|437022.15.peg.1520 | fig\|6666666.707072.peg.1270 |
|  | *alaC -* Glutamate-pyruvate aminotransferase (EC 2.6.1.2) | fig\|437022.15.peg.1021 | *NA* |
|  | *avtA* -Valine-pyruvate aminotransferase (EC 2.6.1.66) | *NA* | *NA* |
|  | *alr* - Alanine racemase (EC 5.1.1.1) | fig\|437022.15.peg.299 | fig\|6666666.707072.peg.407 |
|  |  | **Pathway Asserted** | **Pathway Not Asserted** |
|  | | | |
| **L-Arginine** | *argA* - Amino-acid N-acetyltransferase (EC 2.3.1.1) | *NA* | *NA* |
|  | *argJ -* Arginine biosynthesis bifunctional protein (EC 2.3.1.1) | *NA* | *NA* |
|  | *argB* - Acetylglutamate kinase (EC 2.7.2.8) | *NA* | *NA* |
|  | *argC* - N-acetyl-gamma-glutamyl-phosphate reductase (EC 1.2.1.38) | *NA* | *NA* |
|  | *argD* - Acetylornithine/succinyldiaminopimelate aminotransferase (EC 2.6.1.17) | *NA* | *NA* |
|  | *argF* -N-acetylornithine carbamoyltransferase (EC 2.1.3.9) | *NA* | *NA* |
|  | *argE* - Acetylornithine deacetylase (EC 3.5.1.16) | *NA* | *NA* |
|  | *argG* - Argininosuccinate synthase (EC 6.3.4.5) | fig\|437022.15.peg.847 | fig\|6666666.707072.peg.2198 |
|  | *argH* - Argininosuccinate lyase (EC 4.3.2.1) | fig\|437022.15.peg.846 | fig\|6666666.707072.peg.2197 |
|  | *argI* - Ornithine carbamoyltransferase (EC 2.1.3.3) | *NA* | *NA* |
|  | *carA* - Carbamoyl-phosphate synthase small chain (EC 6.3.5.5) | fig\|437022.15.peg.566 | fig\|6666666.707072.peg.688 |
|  | *carB* - Carbamoyl-phosphate synthase large chain (EC:6.3.5.5) | fig\|437022.15.peg.567 | fig\|6666666.707072.peg.689 |
|  | *argR -* Arginine pathway regulatory protein ArgR | fig\|437022.15.peg.851 | fig\|6666666.707072.peg.2202 |
|  |  | **Pathway Not Asserted** | **Pathway Not Asserted** |
|  | | | |
| **L-Asparagine** | *asnB* -Asparagine synthetase [glutamine-hydrolyzing] (EC 6.3.5.4) | fig\|437022.15.peg.425 fig\|437022.15.peg.1941  fig\|437022.15.peg.1954 | *NA* |
|  | *ansA* - L-asparaginase (EC 3.5.1.1) | fig\|437022.15.peg.113 | *NA* |
|  |  | **Pathway Asserted** | **Pathway Not Asserted** |
|  | | | |
| **L-Aspartate** | *aspB* - Aspartate aminotransferase (EC 2.6.1.1) | fig\|437022.15.peg.384  fig\|437022.15.peg.680  fig\|437022.15.peg.2413 | fig\|6666666.707072.peg.2230  fig\|6666666.707072.peg.2272 |
|  |  | **Pathway Asserted** | **Pathway Asserted** |
|  | | | |
| **L-Cysteine** | *cysE* - Serine acetyltransferase (EC 2.3.1.30) | *NA* | *NA* |
|  | *cysK* - Cysteine synthase A/ O-acetylserine sulfhydrylase A (EC 2.5.1.47) | *NA* | *NA* |
|  | *cysM -* Cysteine synthase B (EC 2.5.1.113) | *NA* | *NA* |
|  | *metK* - S-adenosylmethionine synthetase (EC 2.5.1.6) | fig\|437022.15.peg.159 | fig\|6666666.707072.peg.2883 |
|  | *ahcY* - Adenosylhomocysteinase (EC 3.3.1.1) | fig\|437022.15.peg.158 | fig\|6666666.707072.peg.2884 |
|  | *mccA -* Cystathionine beta-synthase (EC 4.2.1.22) | fig\|437022.15.peg.321 | fig\|6666666.707072.peg.2758 |
|  | *mccB -* Cystathionine gamma-lyase (EC 4.4.1.1) | fig\|437022.15.peg.320 | fig\|6666666.707072.peg.2759 |
|  |  | **Pathway Not Asserted** | **Pathway Not Asserted** |
|  | | | |
| **L-Glutamate** | *gltB* - glutamate synthase [NADPH] large chain (EC 1.4.1.13) | *NA* | *NA* |
|  | *gltB2* - Ferredoxin-dependent glutamate synthase (EC 1.4.7.1) | fig\|437022.15.peg.1560 | *NA* |
|  | *gdhA* - Glutamate dehydrogenase (EC 1.4.1.4) | *NA* | *NA* |
|  | *gdhB -* NAD-specific glutamate dehydrogenase (EC 1.4.1.2) | fig\|437022.15.peg.750 | fig\|6666666.707072.peg.2126 |
|  | *murI* - Glutamate racemase (EC 5.1.1.3) | fig\|437022.15.peg.916 | fig\|6666666.707072.peg.1099 |
|  | *putA -* Proline dehydrogenase (EC 1.5.99.8) (Proline oxidase) / Delta-1-pyrroline-5-carboxylate dehydrogenase (EC 1.5.1.12) | fig\|437022.15.peg.948 | fig\|6666666.707072.peg.1126 |
|  |  | **Pathway Asserted** | **Pathway Asserted** |
|  | | | |
| **L-Glutamine** | *glnA* - Glutamine synthetase (EC 6.3.1.2) | fig\|437022.15.peg.2491 | fig\|6666666.707072.peg.2958 |
|  | *glsA* - Glutaminase (EC 3.5.1.2) | fig\|437022.15.peg.2348 | fig\|6666666.707072.peg.2808 |
|  |  | **Pathway Asserted** | **Pathway Asserted** |
|  | | | |
| **Glycine** | *itaE* - Low specificity L-threonine aldolase (EC 4.1.2.48) | *NA* | fig\|6666666.707072.peg.2156 |
|  | *glyA* - Serine hydroxymethyltransferase (EC 2.1.2.1) | fig\|437022.15.peg.2187 | fig\|6666666.707072.peg.537 |
|  |  | **(Interconversion with Serine)** | **(Interconversion with Serine)** |
|  | | | |
| **L-Histidine** | *hisG* -ATP phosphoribosyltransferase (EC 2.4.2.17) | fig\|437022.15.peg.861 | fig\|6666666.707072.peg.616 |
|  | *hisIE* - Phosphoribosyl-AMP cyclohydrolase (EC 3.5.4.19) / Phosphoribosyl-ATP pyrophosphatase (EC 3.6.1.31) | fig\|437022.15.peg.868 | fig\|6666666.707072.peg.623 |
|  | *priA/hisA -* Phosphoribosylformimino-5-aminoimidazole carboxamide ribotide isomerase (EC 5.3.1.16) | fig\|437022.15.peg.866 | fig\|6666666.707072.peg.621 |
|  | *hisF* - Imidazole glycerol phosphate synthase cyclase subunit (EC 4.3.2.10) | fig\|437022.15.peg.419  fig\|437022.15.peg.867 | fig\|6666666.707072.peg.622  fig\|6666666.707072.peg.2621 |
|  | *hish* - Imidazole glycerol phosphate synthase amidotransferase subunit (EC 4.3.2.10) | fig\|437022.15.peg.420,  fig\|437022.15.peg.865 | fig\|6666666.707072.peg.620  fig\|6666666.707072.peg.2620 |
|  | *hisB -* Histidinol-phosphatase (EC 3.1.3.15) / Imidazoleglycerol-phosphate dehydratase (EC 4.2.1.19) | fig\|437022.15.peg.864 |  |
|  | *hisC* - Histidinol-phosphate aminotransferase (EC 2.6.1.9) | fig\|437022.15.peg.863 | fig\|6666666.707072.peg.618  fig\|6666666.707072.peg.1855 |
|  | *hisN* - Histidinol-phosphatase (EC 3.1.3.15) | fig\|437022.15.peg.7 | fig\|6666666.707072.peg.7  fig\|6666666.707072.peg.619 |
|  | *hisD* - Histidinol dehydrogenase (EC 1.1.1.23) | fig\|437022.15.peg.862 | fig\|6666666.707072.peg.617 |
|  |  | **Pathway Asserted** | **Pathway Asserted** |
|  | | | |
| **L-Isoleucine** | *ilvA* - Threonine deaminase (EC 4.3.1.19) | fig\|437022.15.peg.2073 | *NA* |
|  | *ridA -* 2-iminobutanoate/2-iminopropanoate deaminase (EC 3.5.99.10) | *NA* |  |
|  | *ilvN* - Acetolactate synthase (EC 2.2.1.6)  *ilvBN* - Acetolactate synthase isozyme 1 large/small subunit (EC 2.2.1.6)  ilvGM - Acetolactate synthase isozyme 2 large/small subunit (EC 2.2.1.6)  *ilvIH* - Acetolactate synthase isozyme 3 large/small subunit (EC 2.2.1.6) | *NA* | *NA* |
|  | *ilvC* - Ketol-acid reductoisomerase; NADP^+^ (EC 1.1.1.86) | *NA* | *NA* |
|  | *ilvD* - Dihydroxy-acid dehydratase (EC 4.2.1.9) | *NA* | *NA* |
|  | *ilvE* - Branched-chain-amino-acid aminotransferase (EC 2.6.1.42) | fig\|437022.15.peg.248 | fig\|6666666.707072.peg.339 |
|  |  | **Pathway Not Asserted** | **Pathway Not Asserted** |
|  | | | |
| **L-Leucine** | *ilvN - Acetolactate synthase (EC 2.2.1.6)*  *ilvBN - Acetolactate synthase isozyme 1 large/small subunit (EC 2.2.1.6)* | *NA* | *NA* |
|  | *ilvC* - Ketol-acid reductoisomerase (NADP^+^) (EC 1.1.1.86) | *NA* | *NA* |
|  | *leuA* -2-isopropylmalate synthase (EC 2.3.3.13) | fig\|437022.15.peg.1556 | *NA* |
|  | *leuC/leuD* - 3-isopropylmalate dehydratase large/small subunit (EC 4.2.1.33) | fig\|437022.15.peg.1722  fig\|437022.15.peg.1723 | *NA* |
|  | leuB - 3-isopropylmalate dehydrogenase (EC 1.1.1.85) | fig\|437022.15.peg.1721 | *NA* |
|  | ilvE - Branched-chain-amino-acid aminotransferase (EC 2.6.1.42) | fig\|437022.15.peg.248 | *NA* |
|  |  | **Pathway Not Asserted** | **Pathway Not Asserted** |
|  | | | |
| **L-Lysine** | *lysC* - Aspartokinase (EC 2.7.2.4) | fig\|437022.15.peg.486 | fig\|6666666.707072.peg.2531 |
|  | *asd* - Aspartate-semialdehyde dehydrogenase (EC 1.2.1.11) | fig\|437022.15.peg.1720 | fig\|6666666.707072.peg.2018 |
|  | *dapA* - 4-hydroxy-tetrahydrodipicolinate synthase (EC 4.3.3.7) | fig\|437022.15.peg.312,  fig\|437022.15.peg.1754 | fig\|6666666.707072.peg.2762 |
|  | *dapB* - 4-hydroxy-tetrahydrodipicolinate reductase (EC 1.17.1.8) | fig\|437022.15.peg.562 | fig\|6666666.707072.peg.677 |
|  | *dapD* - 2,3,4,5-tetrahydropyridine-2,6-dicarboxylate N-succinyltransferase (EC 2.3.1.117) | fig\|437022.15.peg.1071 | fig\|6666666.707072.peg.1362 |
|  | *dapC* - N-succinyl-L, L-diaminopimelate aminotransferase (EC 2.6.1.17) | NA | *NA* |
|  | *dapE* - N-succinyl-L, L-diaminopimelate desuccinylase (EC 3.5.1.18) | fig\|437022.15.peg.1070 | fig\|6666666.707072.peg.1361 |
|  | *dapF* - Diaminopimelate epimerase (EC 5.1.1.7) | fig\|437022.15.peg.2533 | fig\|6666666.707072.peg.3006 |
|  | *lysA* - Diaminopimelate decarboxylase (EC 4.1.1.20) | fig\|437022.15.peg.2532 | fig\|6666666.707072.peg.3004 |
|  | *dapH* - 2,3,4,5-tetrahydropyridine-2,6-dicarboxylate N-acetyltransferase (EC 2.3.1.89) | *NA* | *NA* |
|  | *dapX -* Probable N-acetyl-L, L-diaminopimelate aminotransferase (EC 2.6.1.-) | *NA* | *NA* |
|  | *ykuR -* N-acetyldiaminopimelate deacetylase (EC 3.5.1.47) | *NA* | fig\|6666666.707072.peg.914 |
|  | *ddh* - Meso-diaminopimelate D-dehydrogenase (EC 1.4.1.16) | *NA* | *NA* |
|  | *argD* - Acetylornithine/succinyldiaminopimelate aminotransferase (EC 2.6.1.17) | *NA* | *NA* |
|  | *serC* - Phosphoserine aminotransferase (EC 2.6.1.52) | fig\|437022.15.peg.1865 | fig\|6666666.707072.peg.2308 |
|  |  | **Pathway Asserted** | **Pathway Asserted** |
|  | | | |
| **L-Methionine** | lysC - Aspartokinase (EC 2.7.2.4) | fig\|437022.15.peg.486 | fig\|6666666.707072.peg.2531 |
|  | asd - Aspartate-semialdehyde dehydrogenase (EC 1.2.1.11) | fig\|437022.15.peg.1720 | fig\|6666666.707072.peg.2018 |
|  | *thrA/hom* - Homoserine dehydrogenase (EC 1.1.1.3) | fig\|437022.15.peg.1022 | NA |
|  | *metA,* ***metAS*** - Homoserine O-succinyltransferase (EC 2.3.1.46) | *NA* | *NA* |
|  | Homoserine O-acetyltransferase (EC 2.3.1.31) | *NA* | *NA* |
|  | *metB* - Cystathionine gamma-synthase (O-succinylhomoserine(thiol)-lyase / O-succinylhomoserine lyase) (EC 2.5.1.48) | *NA* | *NA* |
|  | *metC* - Cystathionine beta-lyase (EC 4.4.1.13) | *NA* | *NA* |
|  | *metY -* O-acetyl-L-homoserine sulfhydrylase (EC 2.5.1.49) | *NA* | *NA* |
|  | *metH* - cobalamin-dependent methionine synthase (EC 2.1.1.13) | *NA* | *NA* |
|  | *metE* - 5-methyltetrahydropteroyltriglutamate-homocysteine methyltransferase (EC 2.1.1.14) | fig\|437022.15.peg.1652 | *NA* |
|  |  | **Pathway Not Asserted** | **Pathway Not Asserted** |
|  | | | |
| **L-Phenylalanine** | *pheA* - Chorismate mutase (EC 5.4.99.5)/ Prephenate dehydratase (EC 4.2.1.51) | *NA* | *NA* |
|  |  | **Pathway Not Asserted** | **Pathway Not Asserted** |
|  | | | |
| **L-Proline** | *rocD* - Ornithine aminotransferase (EC 2.6.1.13) | fig\|437022.15.peg.189 | fig\|6666666.707072.peg.271 |
|  | *proC* - Pyrroline-5-carboxylate reductase (EC 1.5.1.2) | fig\|437022.15.peg.143 | fig\|6666666.707072.peg.2907 |
|  | *proB* - Glutamate 5-kinase (EC 2.7.2.11) | *NA* | *NA* |
|  | *proA* -Gamma-glutamyl phosphate reductase (EC 1.2.1.41) | *NA* | *NA* |
|  | *ocd* - Ornithine cyclodeaminase (EC 4.3.1.12) | NA | *NA* |
|  |  | **Pathway Not Asserted** | **Pathway Not Asserted** |
|  | | | |
| **L-Serine** | *serA* - D-3-phosphoglycerate dehydrogenase (EC 1.1.1.95) | fig\|437022.15.peg.1336 fig\|437022.15.peg.2541 | fig\|6666666.707072.peg.3012 fig\|6666666.707072.peg.1461 |
|  | *serC* - Phosphoserine aminotransferase (EC 2.6.1.52) | fig\|437022.15.peg.1865 | fig\|6666666.707072.peg.2308 |
|  | *serB* - Phosphoserine phosphatase (EC 3.1.3.3) | *NA* | *NA* |
|  |  | **Interconversion with Glycine** | **Interconversion with Glycine** |
|  | | | |
| **L-Threonine** | *lysC* - Aspartokinase (EC 2.7.2.4) | fig\|437022.15.peg.486 | fig\|6666666.707072.peg.2531 |
|  | *asd* - Aspartate-semialdehyde dehydrogenase (EC 1.2.1.11) | fig\|437022.15.peg.1720 | fig\|6666666.707072.peg.2018 |
|  | *thrA -* Bifunctional aspartokinase/homoserine dehydrogenase (EC 2.7.2.4) | *NA* | *NA* |
|  | *thrA/hom* - Homoserine dehydrogenase (EC 1.1.1.3) | fig\|437022.15.peg.1022 | *NA* |
|  | *thrB* - Homoserine kinase (EC 2.7.1.39) | fig\|437022.15.peg.2556 | *NA* |
|  | *thrC* - Threonine synthase (EC 4.2.3.1) | fig\|437022.15.peg.1023 | *NA* |
|  |  | **Pathway Asserted** | **Pathway Not Asserted** |
|  | | | |
| **L-Tryptophan** | *trpE* - Anthranilate synthase, aminase component (EC 4.1.3.27) | fig\|437022.15.peg.937 | *NA* |
|  | *PabA* - Anthranilate synthase, amidotransferase component (EC 4.1.3.27) | fig\|437022.15.peg.936 fig\|437022.15.peg.763 | fig\|6666666.707072.peg.2110 |
|  | *trpD* - Anthranilate phosphoribosyltransferase (EC 2.4.2.18) | fig\|437022.15.peg.935 | fig\|6666666.707072.peg.1113 |
|  | *trpCF* - Indole-3-glycerol phosphate synthase (EC 4.1.1.48) / Phosphoribosylanthranilate isomerase (EC 5.3.1.24) | fig\|437022.15.peg.934 | fig\|6666666.707072.peg.1112 |
|  | *trpA* - Tryptophan synthase alpha chain (EC 4.2.1.20) | fig\|437022.15.peg.932 | fig\|6666666.707072.peg.1110 |
|  | *trpB* - Tryptophan synthase beta chain (EC 4.2.1.20) | fig\|437022.15.peg.933 | fig\|6666666.707072.peg.1111 |
|  | *pabB* - Para-aminobenzoate synthase, aminase component (EC 2.6.1.85) | fig\|437022.15.peg.1661 | fig\|6666666.707072.peg.1162 |
|  | *priA/hisA* - Phosphoribosylformimino-5-aminoimidazole carboxamide ribotide isomerase (EC 5.3.1.16) | fig\|437022.15.peg.866 | fig\|6666666.707072.peg.621 |
|  |  | **Pathway Asserted** | **Pathway Asserted** |
|  | | | |
| **L-Tyrosine** | *pheA -* Chorismate mutase (EC 5.4.99.5)/ Prephenate dehydratase (EC 4.2.1.51) | *NA* | *NA* |
|  | *tyrB* - Aromatic-amino-acid aminotransferase (EC:2.6.1.57) | fig\|437022.15.peg.349 |  |
|  |  | **Pathway Not Asserted** | **Pathway Not Asserted** |
|  | | | |
| **L-Valine** | *ilvN* - Acetolactate synthase (EC 2.2.1.6)  *ilvBN* - Acetolactate synthase isozyme 1 large/small subunit (EC 2.2.1.6)  *ilvGM* - Acetolactate synthase isozyme 2 large/small subunit (EC 2.2.1.6)  *ilvIH* - Acetolactate synthase isozyme 3 large/small subunit (EC 2.2.1.6) | *NA* | *NA* |
|  | *ilvC* - Ketol-acid reductoisomerase (NADP^+^) (EC 1.1.1.86) | *NA* | *NA* |
|  | *ilvD* - Dihydroxy-acid dehydratase (EC 4.2.1.9) | *NA* | *NA* |
|  | *ilvE* - Branched-chain-amino-acid aminotransferase (EC 2.6.1.42) | fig\|437022.15.peg.248 | fig\|6666666.707072.peg.339 |
|  |  | **Pathway Not Asserted** | **Pathway Not Asserted** |
