# Supplementary material for: The inside scoop: Comparative genomics of two intranuclear bacteria, “Candidatus Berkiella cookevillensis” and “Candidatus Berkiella aquae”
Source: PLoS One. 2022 Dec 30;17(12):e0278206. doi: 10.1371/journal.pone.0278206 (PMC9803151; doi:10.1371/journal.pone.0278206)
Supplement: S1 Text — (DOCX) [file pone.0278206.s006.docx]

**S1 Text Amino acid biosynthesis pathways of “*Ca*. B. cookevillensis” (CC99) and “*Ca*. B. aquae” (HT99)**

Both CC99 and HT99 may be capable of synthesizing L-glutamate and L-glutamine, major amino donors for several amino acids. Genes for NAD-specific glutamate dehydrogenase (*gdhB*) and glutamine synthetase (*glnA*) were identified in both bacteria. Glutamate dehydrogenase catalyzes the reversible oxidative deamination of L-glutamate to α–ketoglutarate and ammonia while glutamine synthetase incorporates ammonia into glutamate to form glutamine. CC99 also encodes a gene for a ferredoxin-dependent glutamate synthase (*gltB2*), an enzyme that synthesizes L-glutamate from α-ketoglutarate and L-glutamine. Genes for glutaminase (an enzyme which generates L-glutamate from L-glutamine) and glutamate racemase (an enzyme which converts L-glutamate to D-glutamate) are also present in both bacteria.

Both bacteria encode aspartate aminotransferase (*aspB*), an enzyme that catalyzes a transamination reaction that converts oxaloacetate to L-aspartate, suggesting a capability of L-aspartate biosynthesis (Fig 4, S2 Table). They also encode aspartate kinase (*lysC*) and aspartate-semialdehyde dehydrogenase (*asd*) which convert L-aspartate to L-aspartate-4-semialdehyde, a branch point for a pathway that leads towards biosynthesis of L-lysine and another branch that leads to the biosynthesis of L-threonine and L-methionine [1]. Genes encoding enzymes that catalyze the conversion of L-aspartate-4-semialdehyde to L-2,3,4,5-tetrahydrodipicolinate (THDP; *dapA, dapB*), THDP to meso-diaminopimelate (m-DAP; *dapD, serC, dapE, dapF,*) and conversion of m-DAP to L-lysine (l*ysA*) were identified in both bacteria (fig. 4, table S2). Thus, these bacteria may be capable of synthesizing L-lysine from L-aspartate. The L-threonine biosynthesis pathway appears to be intact in CC99. HT99, however, is missing genes encoding key enzymes involved in the L-threonine biosynthesis pathway, including homoserine dehydrogenase (*thrA/hom*), homoserine kinase (*thrB*), and threonine synthase (*thrC*). A *de novo* pathway for L-methionine biosynthesis was not identified in both bacteria.

L-serine synthesized from 3-phosphogylcerate (an intermediate of glycolysis) is an important precursor for the biosynthesis of glycine and L-cysteine. Both bacteria have genes encoding D-3-phosphoglycerate dehydrogenase (*serA*) and phosphoserine aminotransferase (*serC*), which catalyze the oxidation of 3-phosphogylcerate to phosphohydroxypyruvate and the subsequent conversion of phosphohydroxypyruvate to phosphoserine, respectively (Fig 4, S2 Table). However, both bacteria are missing the gene for phosphoserine phosphatase (*serB*) responsible for hydrolysis of phosphate from phosphoserine, so they may not be able to synthesize L-serine via this pathway. However, a gene for serine hydroxymethyltransferase (*glyA*), an enzyme which catalyzes the interconversion of L-serine and glycine, was identified in both bacteria. A gene encoding L-threonine aldolase (*itaE*) was also identified in HT99 but not in CC99. L-threonine aldolase catalyzes the cleavage of L-threonine to generate glycine and acetaldehyde.

Genes encoding for enzymes involved in the biosynthesis of L-histidine from PRPP (derived from ribose-5-phosphate) are present (Fig 4, S2 Table). Genes encoding enzymes involved in a two-step conversion of L-serine to L-cysteine via O-acetyl-L-serine were not identified in either bacterium. However, both bacteria have genes encoding cystathionine beta-synthase (*mccA*) and cystathionine gamma-lyase (*mccB*) involved in the conversion of L-homocysteine to L-cysteine via the reverse trans-sulfuration pathway [2]. The gene for cysteine desulfurase (*iscS*), an enzyme which catalyzes the conversion of L-cysteine (if available as a source) to L-alanine, was identified in both bacteria (Fig 4, S2 Table). CC99 may be capable of synthesizing L-alanine from pyruvate as it has a gene encoding glutamate-pyruvate aminotransferase (*alaA*), an alanine synthesizing transaminase. A gene for asparagine synthetase (*asnB*), an enzyme that is catalyzes the ATP-dependent conversion of L-aspartate into L-asparagine, was identified in CC99 but not in HT99.

Genes for key enzymes involved in the biosynthesis of L-arginine and L-proline are absent in both bacteria, including enzymes involved in the synthesis of N(2)-acetyl-L-ornithine (*argA, argB, argC, argD*) and L-glutamate 5-semialdehyde (*proA, proB*) from L-glutamate (Fig 4, S2 Table). Both bacteria encode pyrroline-5-carboxylate reductase *(proC*), responsible for conversion L-glutamate-5-semialdehyde to L-proline.

Genes involved in the synthesis of L-leucine from 3-methyl-2-oxobutanoate were identified in CC99 but not in HT99. Both bacteria are missing several genes encoding enzymes involved in the synthesis of L-isoleucine and L-valine and so may not be capable of synthesizing these branched chain amino acids. Genes encoding enzymes that convert erythrose 4-phosphate to chorismic acid, an important precursor for the aromatic amino acids L-phenylalanine, L-tryptophan, and L-tyrosine, are present in both bacteria. The genes encoding enzymes responsible for the formation of L-tryptophan from chorismic acid were identified arranged in a single cluster in both bacteria. Both bacteria, however, lack genes encoding enzymes involved in converting chorismate to L-phenylalanine and L-tyrosine (Fig 4, S2 Table).

**References**

1. Davis BD. Diaminopimelic Acid and Lysine: Biosynthetic Interrelations of Lysine, Diaminopimelic Acid, and Threonine in Mutants of *Escherichia coli*. Nature. 1952 Mar 1;169(4300):534–6.

2. Hullo MF, Auger S, Soutourina O, Barzu O, Yvon M, Danchin A, et al. Conversion of methionine to cysteine in *Bacillus subtilis* and its regulation. J. Bacteriol. 2007 Jan 1;189(1):187.
